# Self-Assembly Supramolecular Systems Based on Guanidinium Salts Modified Hyperbranched Polyamidoamine and Cationic Acrylamide Copolymers

**DOI:** 10.3390/polym11111781

**Published:** 2019-10-31

**Authors:** Qi Wu, Shaohua Gou, Yumei Fei, Xiaoyan Yang, Mengyu Liu, Jinglun Huang

**Affiliations:** 1 College of Chemistry and Chemical Engineering, Southwest Petroleum University, Chengdu 610500, China; 201722000166@swpu.stu.edu.cn (Q.W.); 201621000271@swpu.stu.edu.cn (Y.F.); 201721000259@swpu.stu.edu.cn (X.Y.); 201722000183@swpu.stu.edu.cn (M.L.); 2 Institute of Chemical Materials, China Academy of Engineering Physics, Mianyang 621900, China; jinglunhuang@163.com

**Keywords:** self-assembly supramolecular systems, Guanidinium Salts Modified Hyperbranched Polyamidoamine, cationic acrylamide copolymers, clay hydration inhibition

## Abstract

Herein, novel hyperbranched polyamidoamine guanidinium salts (GS-h-PAMAM) and two cationic acrylamide copolymers P(AM-DAC-ABSM) and P(AM-DAC-AMTU) were successfully prepared. Then, self-assembly supramolecular systems were synthesized by directly mixing GS-h-PAMAM with copolymers in aqueous solution, and the mechanism of the self-assembly process was speculated. FT-IR, NMR, and SEM were used for structural confirmation. Furthermore, the excellent solution properties revealed that the supramolecular systems had potential application in clay hydration inhibitors. More importantly, utilizing functionalized hyperbranched polyamidoamine in the synthesis self-assembly supramolecular systems was an effective strategy for expanding their application fields and developing new functional materials, providing a powerful reference for the next study.

## 1. Introduction

**Figure 1:**
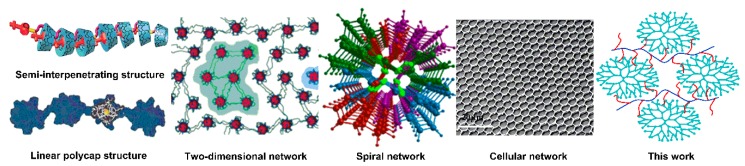
The typical supramolecular systems with different structures.

The supramolecular system was composed of two or more molecules through a non-covalent bond force such as hydrogen bonding [1], π-π stacking [2,3], hydrophilic (hydrophobic) interaction [4], ionic interaction [5,6] anion template [7], etc. Recently, supramolecular systems based on host-guest interaction [8,9,10] and self-assembly [11,12] had been successfully prepared and showed potential applications in many aspects (Figure 1).

Hyperbranched polyamidoamine, which was synthesized by a simple one-pot method, overcame the cumbersome and costly defects in the preparation and separation of dendritic polyamide amines. In particular, the self-assembly supramolecular system based on hyperbranched polyamidoamine had become a wide topic [11,13,14,15]. Liu et al. [11] reported a preparation method of amphiphilic hyperbranched polyamidoamine fluorescent honeycomb-patterned films, presented and validated a supramolecular complex self-assembly strategy for the facile fabrication of multifunctional thin films with ordered microporous patterns, which greatly enlarged the diversity of structures that could be assembled, opened up a straightforward route to the functionalization of self-assembly structures (Figure 1). Sun et al. [15] synthesized a novel hyperbranched polyamidoamine based self-assembly supramolecular polymeric micelle EGCG–HPAM–Dex via electrostatic, hydrogen bonding and hydrophobic interactions. The MTT assay revealed that the HPAM–Dex conjugates had lower cytotoxicity than pristine HPAM owing to the introduction of dextran. In addition, the stability and the drug loading and release behaviors of the EGCG–HPAM–Dex micelles were studied. In fact, the presence of large numbers of amino groups and cavities caused easier generation of high-density hydrogen bonds and Coulomb forces in the molecule, which promoted the achievement of self-assembly. Moreover, the polymer molecular also had the self-fluorescent performance [16,17]. Due to these unique properties, the hyperbranched polyamidoamine was expected to be used in many files, such as biomedicine [18,19,20,21,22], nanomaterials [23,24,25], sensing [26,27], etc. However, it was not limited to these.

As we all know, functionalization of hyperbranched polymers was a reliable way to expand their application fields and develop new functional materials [28,29]. Nevertheless, there was less study on the preparation and performance of functionalized hyperbranched polyamidoamine based self-assembly supramolecular systems. In this work (Figure 1), a novel hyperbranched polyamidoamine guanidinium salts (GS-h-PAMAM) was synthesized via addition reaction of dicyandiamide and hyperbranched polyamidoamine (h-PAMAM). And two cationic acrylamide copolymers P(AM-DAC-ABSM) and P(AM-DAC-AMTU) were successfully prepared via solution radical polymerization. Then, self-assembly supramolecular systems were prepared by directly mixing GS-h-PAMAM with copolymers in aqueous solution. FT-IR, NMR, and SEM were used for structural confirmation, and we also speculated the mechanism of the self-assembly process. Furthermore, the solution properties of supramolecular systems were comparatively studied with corresponding copolymer systems.

## 2. Experimental

### 2.1. Materials

All chemical regents used in this research were analytical grade materials (ChengDu Chron Chemicals, Chengdu, China, and Aladdin, Ontario, CA, USA) without further purification.

### 2.2. Instrumentation and Analytical Methods

FT-IR spectra was recorded on a Fourier transform Infrared Spectrometer (WQF-520, Ruilian, China) in the range of 4000–500 cm^−1^, ^1^H NMR spectra was recorded on a BRUKER AV III 400 MHz Nuclear Magnetic Resonance Spectrometer (Bruker Co., Fällanden, Switzerland). SEM was observed via a scanning electron microscope (FEI Quanta 450, Hillsboro, OR, USA). Copolymer composition was determined by HPLC (LC-20A, Hitachi, Tokyo, Japan). Thermal stability was tested via a Thermogravimetric Analyzer (METTLER TOLEDO Co., Switzerland) at a scan rate of 40 °C·min^−1^ under nitrogen atmosphere of 20 mL·min^−1^. Rheological behavior was conducted by a HAAKE MARS III Rheometer (HAAKE, Vreden, Germany). Centrifugal operation by a Centrifuge (TDL-4, Anting, China), cuttings recovery experiment via a Variable Frequency Roller Heating Furnace (BRGL-7, Tongchun, China).

### 2.3. Synthetic Methods

#### 2.3.1. Preparation of Copolymer P(AM-DAC-ABSM)

Certain amounts of acrylamide (AM), acryloyloxyethyltrimethylammonium chloride (DAC), allyl sulfonamide (ABSM), emulsifier OP-10 (0.5 mL) and 20 mL deionized water were added together into a 100 mL three-necked round bottom flask with a magnetic stir bar, stirred until the solid was completely dissolved. Then nitrogen gas was introduced at a certain temperature for 15 min, a certain amount of initiator azobisisobutylphosphonium hydrochloride (V_50_) was added and kept nitrogen gas atmosphere for 10 min, the system was kept at a constant temperature for 8 h. Then the crude product was repeatedly purified by using ethanol, pulverized, and dried under vacuum at 45 °C for 72 h, a white solid powder P(AM-DAC-ABSM) was obtained (Feeding and optimization details see the ESI).

FT-IR (cm^−1^): 3430(vs), 2950(m), 2120(w), 1680(vs), 1450(s), 1158(s), 940(s).

#### 2.3.2. Preparation of Copolymer P(AM-DAC-AMTU)

Certain amounts of acrylamide (AM), acryloyloxyethyltrimethylammonium chloride (DAC), 1-allyl-3-methylthiourea (AMTU) and 20 mL deionized water were added together into a 100 mL three-necked round bottom flask with a magnetic stir bar, stirred until the solid was completely dissolved. Then nitrogen gas was introduced at a certain temperature for 15 min, a certain amount of initiator azobisisobutylphosphonium hydrochloride (V_50_) was added and kept nitrogen gas atmosphere for 10 min, the system was kept at a constant temperature for 8 h. Then the crude product was repeatedly purified by using ethanol, pulverized, and dried under vacuum at 45°C for 72 h, a white solid powder P(AM-DAC-ABSM) was obtained (Feeding and optimization details see the ESI).

FT-IR (cm^−1^): 3510(vs), 2930(s), 2850(w), 2145(m), 1695(w), 1590(vs), 1345(vs).

#### 2.3.3. Preparation of h-PAMAM

The h-PAMAM was prepared according to the literature methods [30]. A certain amount of diethylene triamine (DETA) dissolved with 20 mL methanol was added into a 100 mL single-necked round bottom flask with a magnetic stir bar, the system was kept nitrogen gas atmosphere for 20 min, then a certain amount of methyl acrylate (MA) was added with stirring, the mixture was reacted at 25 °C for 24 h, evaporated to remove the solvent methanol, the remained products were continued to react at a certain temperature for 12 h. Then the reaction system was cooled to room temperature, the crude product was dissolved with methanol, and precipitated with diethyl ether, filtered, filter cake was washed with diethyl ether three times and dried under vacuum, a light yellow product h-PAMAM was obtained (Feeding and optimization details see the ESI).

^1^H NMR (400 MHz, D_2_O) δ (ppm): 3.25-3.52 (–C(O)–NH–C**H**_2_–), 2.38-2.78 (–C**H**_2_–NH–and–C**H**_2_–NH_2_), 1.10-1.16 (–N**H**– and –N**H**_2_).

FT-IR (cm^−1^): 3440(vs), 2850(s), 1660(s), 1560(w), 1460(w), 1040(m).

#### 2.3.4. Preparation of GS-h-PAMAM

Certain amounts of h-PAMAM, dicyandiamide (DCDA) and 50 mL deionized water were added into a 250 mL three-necked round bottom flask with a magnetic stir bar, then raised the system temperature refluxed for 4 h, the pH of the reaction system was controlled with 1 mol/L hydrochloric acid during the whole process. When the reaction was finished, cooled the mixture to room temperature, a large amount of crystals was precipitate with low temperature atmosphere, filtered, washed the crystals with absolute ethanol and dried under vacuum, then a white crystal product GS-h-PAMAM was obtained (Feeding and optimization details see the ESI).

FT-IR (cm^−1^): 3430(s), 3150(s), 2880(w), 2130(vs), 1660(vs), 1570(m), 1495(m), 1060(s).

## 3. Results and Discussion

### 3.1. Synthesis and Characterization

#### 3.1.1. Monomers and Copolymers

**Scheme 1:**
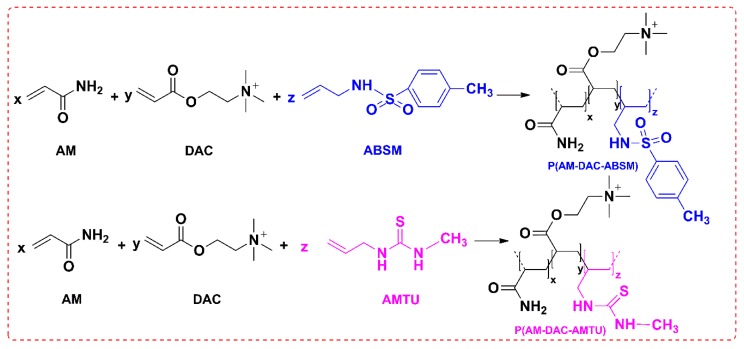
Synthetic routes of P(AM-DAC-ABSM) and P(AM-DAC-AMTU).

The synthesis of monomers ABSM and AMTU were based on Hinsberg and nucleophilic addition reaction, respectively (Figures S1 and S3). The structures were confirmed through FT-IR and ^1^H NMR spectra (Figures S2 and S4). Copolymers P(AM-DAC-ABSM) and P(AM-DAC-AMTU) were prepared via solution radical polymerization (Scheme 1), the optimization towards preparation conditions were carried out (Figures S5 and S6) and the optimum conditions were shown in Table 1. In addition, FT-IR spectra provided strong evidence for the successful synthesis of copolymers (Figure S7), HPLC was utilized as a powerful tool to determine the polymer composition and monomer conversion (Tables S1 and S2). We also performed the measurement of intrinsic viscosity (Figure S8) and analysis of thermal stability (Figure S9). The results indicated the copolymers were successfully prepared and with a good performance (all above details see the Supporting Information).

#### 3.1.2. GS-h-PAMAM and h-PAMAM

**Scheme 2:**
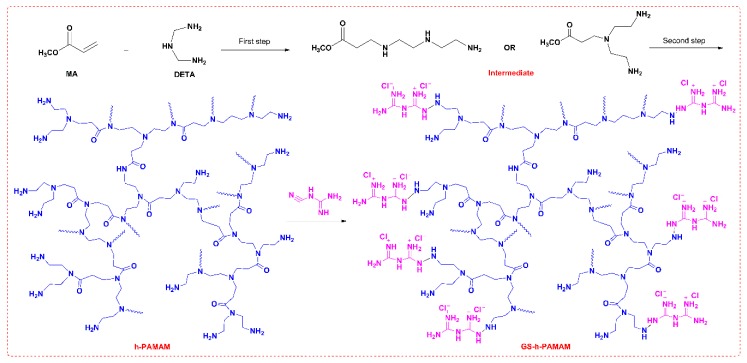
Synthetic routes of h-PAMAM and GS-h-PAMAM.

**Figure 2:**
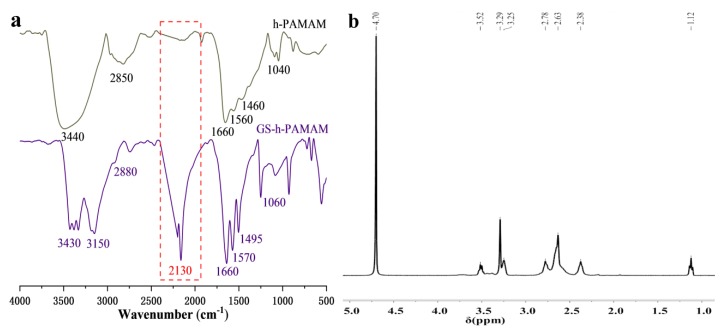
(**a**) FT-IR of h-PAMAM and GS-h-PAMAM; (**b**) ^1^H NMR of h-PAMAM.

**Figure 3:**
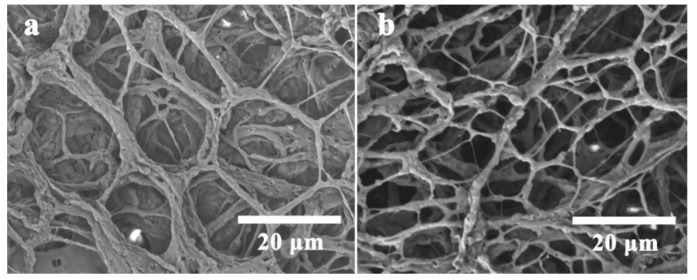
SEM of (**a**) h-PAMAM and (**b**) GS-h-PAMAM.

Hyper-branched polyamidoamine (h-PAMAM) was prepared via a simple two-step reaction (Scheme 2), the first step was a Michael addition reaction between a double bond in methyl acrylate and a primary or secondary amino group on diethylenetriamine to form two corresponding reaction intermediates, the second step was a random amidation reaction at elevated temperatures from the amino and ester groups between the intermediates to give h-PAMAM. Hyperbranched polyamidoamine guanidinium salts (GS-h-PAMAMs) were synthesized by addition reaction of dicyandiamide with amino end group of h-PAMAM (Scheme 2). Also, we optimized the preparation conditions of h-PAMAM and GS-h-PAMAM (Tables S3 and S4, details see the ESI) and displayed the optimum conditions in Table 2. FT-IR and ^1^H NMR were used for structural confirmation (Figure 2). For h-PAMAM, peaks at 3.25–3.52 ppm in the ^1^H NMR spectrum were assigned to the proton peaks of the –C(O)–NH–C***H***_2_– groups, the proton peaks of –C***H***_2_–NH– and –C***H***_2_–NH_2_ bonds were observed at 2.38–2.78 ppm, peaks at 1.10–1.16 ppm were attributed to –NH– and –NH_2_ groups. The strong absorption peak at 3440 cm^−1^ was due to N–H bonds of –NH_2_ and –NH– groups, the C–H bonds of –CH_2_– groups was observed at 2850 cm^−1^, the peaks at 1660, 1560, and 1460 cm^−1^ were due to C=O bonds at different chemical environments, the characteristic peaks at 1040 cm^−1^ confirmed the presence of C–N groups. For GS-h-PAMAM, the structure was similar to h-PAMAM except that the amino end group in the reaction to form –C=NH_2_^+^, so it was only necessary to determine whether its structure contained a characteristic functional group of C=NH_2_^+^, the absorption peaks at 2130 cm^−1^ in FT-IR spectrum just confirmed this. SEM of h-PAMAM and GS-h-PAMAM were shown in Figure 3. The three-dimensional pores network visually confirmed the formation of hyperbranched polymer structure, in which the GS-h-PAMAM with more dense network.

#### 3.1.3. Self-Assembly Supramolecular Systems

**Figure 4:**
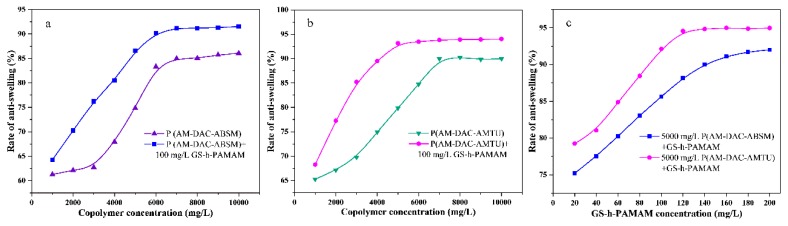
The optimization of supramolecular systems: (**a**) P(AM-DAC-ABSM) concentration; (**b**) P(AM-DAC-ABSM) concentration; (**c**) GS-h-PAMAM concentration.

**Figure 5:**
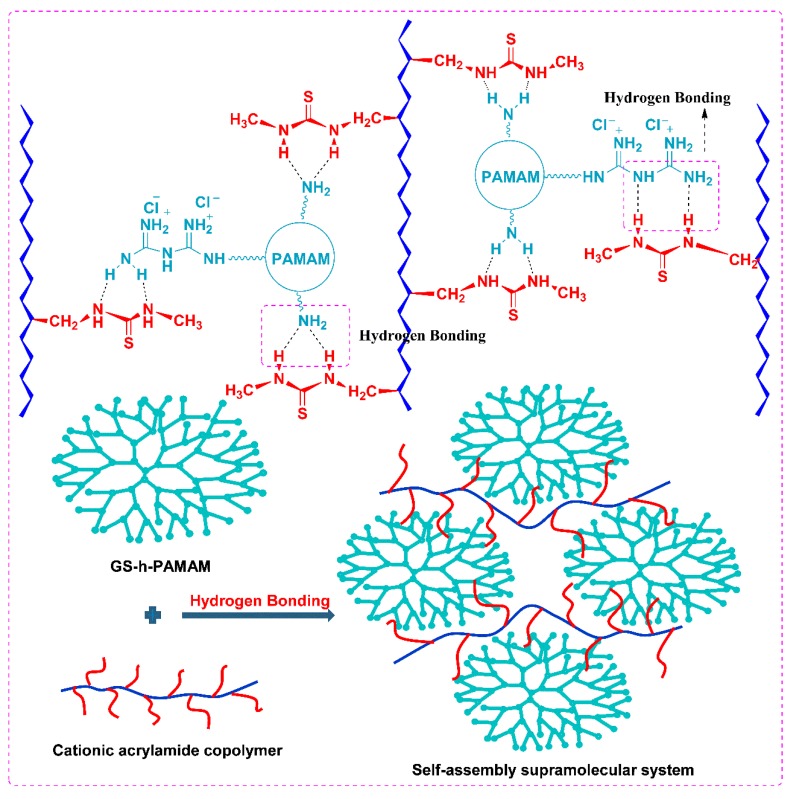
Mechanism of action of GS-h-PAMAM and P(AM-DAC-ATMU) in the self-assembly supramolecular system.

The self-assembly supramolecular system was implemented via simple method. Firstly, a certain amount of copolymer was completely dissolved in deionized water, then a proportion of GS-h-PAMAM were added, after stirred at room temperature for 2 h, an uniform composite system was obtained. The system optimization was based on the effect of composite solutions with different concentrations GS-h-PAMAM and copolymers on the rate of anti-swelling (details see the ESI) of montmorillonite (Figure 4), the optimal supramolecular system was 5000 mg/L copolymer with 120 mg/L GS-h-PAMAM. From Figure 4a,b, there was a general phenomenon that the rate of anti-swelling presented a trend of increased first and then stabilized with the increase of the copolymers and supramolecular systems concentrations, and the addition of GS-h-PAMAM had a significant improvement on the rate of anti-swelling. Interestingly, for copolymer P(AM-DAC-ATMU), the addition of GS-h-PAMAM reduced its concentration (from 7000 mg/L to 5000 mg/L) when the system reached maximum rate of anti-swelling, which was not obvious in P(AM-DAC-ABSM) supramolecular system. For Figure 4c, with the increase of GS-h-PAMAM concentration, the rate of anti-swelling of P(AM-DAC-ATMU) supramolecular system increased and stabilized took 120 mg/L as a turning point. More important, the curve slope of P(AM-DAC-ATMU) supramolecular system gradually increased when the concentration below 120 mg/L, while the P(AM-DAC-ABSM) supramolecular system showed an approximately linear increase. Combined with the results of Figure 4a,b, we thought that GS-h-PAMAM had a significant interaction with copolymer P(AM-DAC-ATMU), but for P(AM-DAC-ABSM) system, the rate of anti-swelling was just a simple superposition of P(AM-DAC-ABSM) and GS-h-PAMAM. Therefore, we speculated the mechanism of action of GS-h-PAMAM and cationic acrylamide copolymers in the self-assembly supramolecular systems (Figure 5). P(AM-DAC-ABSM) and P(AM-DAC-ATMU) were typical cationic acrylamide copolymers, which were mostly linear structures with low molecular weight and weak interaction between molecular chains [31]. Compared to P(AM-DAC-ABSM), the copolymer P(AM-DAC-AMTU) molecular chains contained the thiourea functional group, the special structure similar to “molecular tweezers” could form stable interactions with guest molecules that were prone to form hydrogen bonds [10]. The GS-h-PAMAM contained a plurality of primary amino groups except the guanidinium salts cationic groups, so it could form stable hydrogen bond interactions with the thiourea functional groups of copolymer P(AM-DAC-AMTU), further formed a supramolecular network. In the performance studies in our work, we chose 5000 mg/L copolymer with 100 mg/L GS-h-PAMAM compound systems instead of the optimal systems in order to clearly observe the influence of different conditions on the rate of anti-swelling.

### 3.2. Solution Properties of Copolymers and Supramolecular Systems

#### 3.2.1. Effect of Inorganic Salts

**Figure 6:**
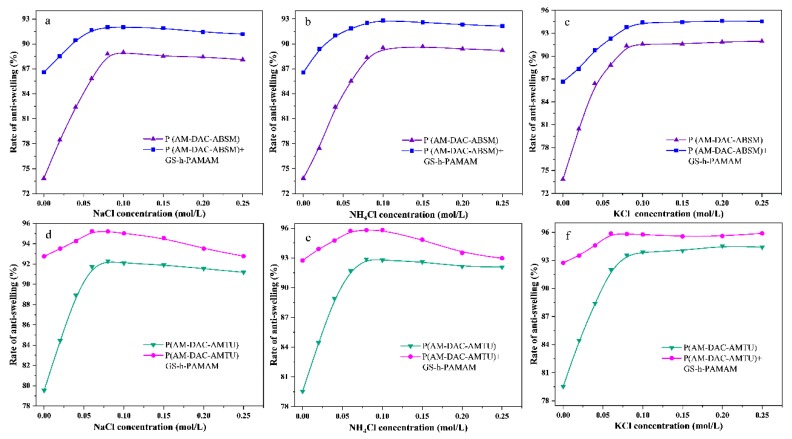
Effect of inorganic salts on rate of anti-swelling: (**a**–**c**) P(AM-DAC-ABSM) and its supramolecular system; (**d**–**f**) P(AM-DAC-AMTU) and its supramolecular system.

The cationic polymer could be adsorbed on the surface of the clay particles to form a dense “protective film”, which prevented and slowed the entry of water molecules into the clay particles. In practical applications, it was found that the inorganic salts could undergo lattice substitution on the clay particle layer and compress the electric double layer, combining with the bridging and coating action of the cationic polymers, which greatly improved the inhibition effect on the hydration expansion of clay particles. Studies also shown that the combination of inorganic salts and polymers had a synergistic effect, caused the increase of the polymer adsorption amount, thereby improving the overall inhibitory effect [32,33]. Therefore, we studied the effects of different concentrations of commonly used inorganic salts (NaCl, NH_4_Cl, and KCl) on the rate of anti-swelling of the solution systems (5000 mg/L copolymers or 5000 mg/L copolymers with 100 mg/L GS-h-PAMAM). As shown in Figure 6, the addition of three inorganic salts significantly improved the rate of anti-swelling of the system, and best effect of KCl with an anti-swelling rate of 96%. The overall trend on rate of anti-swelling was firstly, growth, and then stability with the increase of the concentrations of the salts, and the supramolecular systems were always greater than single copolymer systems, but there were some differences. The curves in Figure 6d,e showed that there was a slight decrease on the rate of anti-swelling when the salts concentration increased to a certain extent, which due to the addition of larger amounts of sodium chloride and ammonium chloride destroyed the hydrogen bonding between copolymer P(AM-DAC-AMTU) and guanidinium salts. Meanwhile, the high salt concentrations also wrecked the molecular chains of the copolymer, as well as undermining the interactions between the molecular chains and the guanidinium salts [34]. Specially, due to the excellent anti-swelling property of KCl itself [35], the loss of the rate of anti-swelling came from the weakening of the interactions due to the increase of the salt concentrations was almost offset, so the above phenomenon was not obvious. As for copolymer P(AM-DAC-AMTU) system, the insignificant decline on rate of anti-swelling was only come from the breaking of molecular chains by inorganic salts because there was no hydrogen bonding.

#### 3.2.2. Effect of Temperature

**Figure 7:**
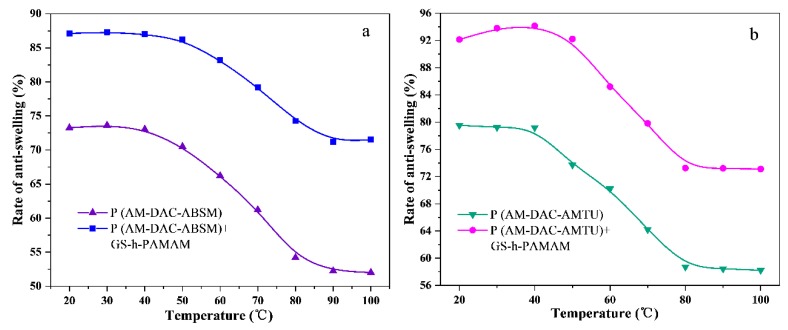
Effect of temperature on the rate of anti-swelling: (**a**) P(AM-DAC-ABSM) and its supramolecular system; (**b**) P(AM-DAC-AMTU) and its supramolecular system.

Temperature is a vital factor affecting the properties of polymer solutions, the molecular chain of acrylamide polymers tends to curl and hydrolyze at high temperatures, resulting in a significant change in the properties of their solutions [36]. In this paper, we studied the relationship between temperature and the rate of anti-swelling of copolymers and corresponding composite solutions. According the results in Figure 7, the rate of anti-swelling of copolymers were gradually reduced and stabilized as the temperature increased, the P(AM-DAC-ABSM) with 19 percentage points declined lower than that with 21 percentage points of P(AM-DAC-AMTU). The better temperature resistance of the copolymer P(AM-DAC-ABSM) benefited from the introduction of a benzene ring rigid structure on its molecular chain [37]. In addition, there was a rising trend as the temperature at 20–40 °C in the P(AM-DAC-AMTU) supramolecular system, the increase of temperature accelerated the thermal movement of copolymer molecular chains and guanidinium salts molecules, and the strong interaction promoted the anti-swelling effect of the supramolecular system. As the temperature further increased, caused the curled of copolymer molecular chains so that cannot effectively wrap clay particles, resulting in a decrease in its own rate of anti-swelling, and at the same time affected the hydrogen bonding with the guanidinium salts, which also led to a decrease in the rate of anti-swelling of the supramolecular system [38]. More, high temperatures promoted the thermal motion of water molecules, thereby accelerating the rate of water entire into the clay particles crystal layers, resulting in a low rate of anti-swelling retention at high temperatures [39].

#### 3.2.3. Effect of Scouring Times

**Figure 8:**
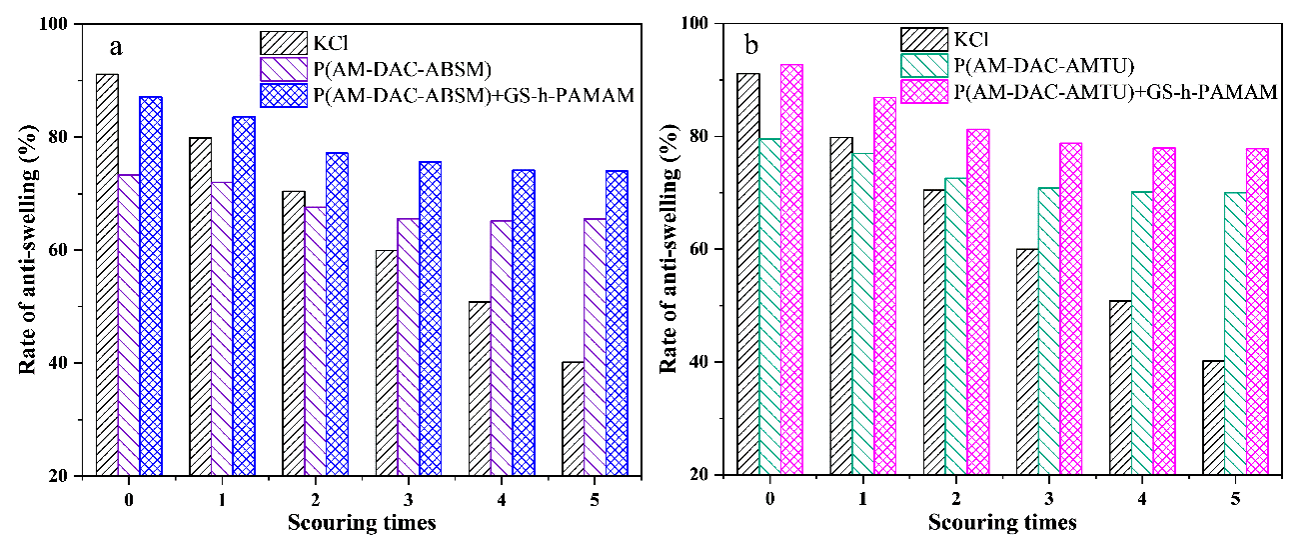
Effect of scouring times on rate of anti-swelling: (**a**) KCl, P(AM-DAC-ABSM) and its supramolecular system; (**b**) KCl, P(AM-DAC-AMTU) and its supramolecular system.

For the reservoirs developed by water injection, long-term water injection was required after pre-treatment with the clay stabilizer solution, and there was also much formation water in the formation, so the anti-swelling agent was required with a certain anti-scouring ability [40]. In this work, we tested the rate of anti-swelling retention of copolymers and corresponding supramolecular systems after different times of scouring (details see the ESI), and compared with the best inorganic salt anti-swelling agents KCl. As shown in Figure 8, after five times of deionized water scouring, the rate of anti-swelling of KCl solution continued to decrease, while the copolymers and corresponding supramolecular solutions with guanidinium salts decreased at first three scouring times and then stabilized with a rate of anti-swelling of 77.8%, and with similar degree of declining. A small-molecule inorganic salt anti-swelling agent, such as KCl, mainly exerted anti-swelling effect by diffusing into the crystal layer of clay particles to reduce the electric double layer repulsive force between the lamellar structures of the clay particles, and had excellent anti-swelling property [41]. However, after washed in multiple times, most of the K^+^ was taken away, which caused the continuous decline in rate of anti-swelling with the increase of scouring times, in agreement with the experimental results. For our copolymers and supramolecular systems, the cationic groups on the polymer chains via multiple points adsorption on the clay particles surface to neutralize the negative charge so that to form a protective film [42], and the hydrophobic groups could also form hydrophobic films on the surface [43], which caused both bridge the clay particles each other and prevent water molecules from diffusing into the layer of clay particles, effectively controlled the hydration dispersion and migration [44].

#### 3.2.4. Effect on Cuttings Recovery Rate

**Figure 9:**
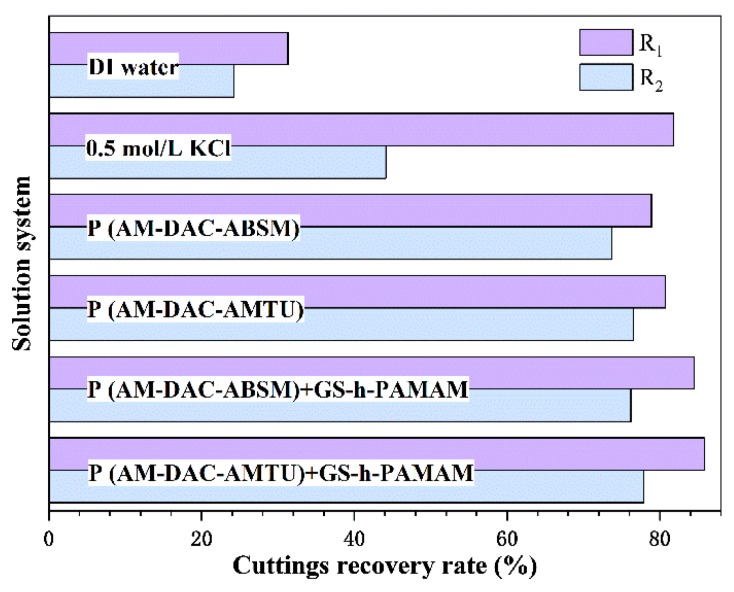
Cuttings recovery rate of different systems.

The cuttings rolling recovery test (details see the ESI) is the most commonly used method to directly evaluate the dispersibility of cuttings in anti-swelling agents, which is a dynamic test conducted under simulated downhole temperature and annulus shear rate, via comparing the recovery of cuttings to measure the stability of different solutions to cuttings [45]. The larger first recovery rate R_1_, the smaller the degree of rock chip dispersion and the better the anti-swelling performance, the larger second recovery rate R_2_, the more stable the anti-swelling effect and the longer the action time. According to Figure 9, after the first hot rolling, there was no obvious difference in cuttings recovery rate R_1_ except the Deionized water system, but significant differences appeared after the second hot rolling. Intuitively, the cuttings particles was best dispersed in deionized water caused the lowest recovery rate, the recovery rate of 0.5 mol/L KCl system was drastically reduced after the second hot rolling due to the poor anti-scourability and short duration of action. More importantly, interesting results were observed that the difference between R_1_ and R_2_ of P(AM-DAC-AMTU) system was lower than the other copolymer system even the two supramolecular systems. The lower decrease amplitude of recovery rate revealed that the copolymer P(AM-DAC-AMTU) had better adsorption stability and longer acting time. For supramolecular systems, the high hot rolling temperature destroyed the hydrogen bonding between the copolymers and the guanidinium salts and increases the water solubility of the guanidinium salts, which caused the reduce of compound effect and the loss of larger amounts of guanidinium salts molecular, resulting in an obvious decrease in the cuttings recovery rate [46].

### 3.3. Rheological Properties

In oilfield applications, polymer solutions are always in constant flow and subjected to external forces [47], so the study of rheological behavior can provide a theoretical basis for their practical applications. In this work, we studied the rheological behavior (details see the ESI) of copolymers and their compound solution systems at 30°C with apparent viscosity as an index.

#### 3.3.1. Shear Thinning

**Figure 10:**
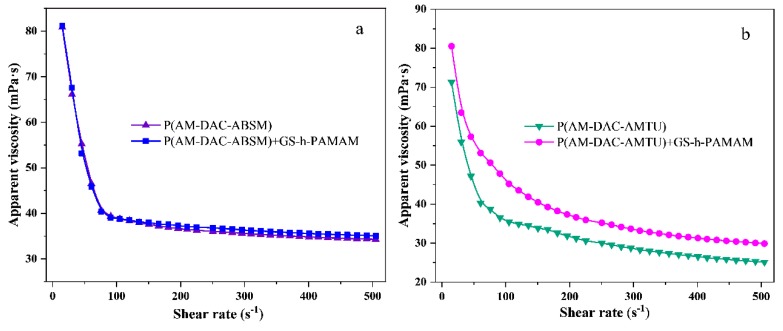
Shear thinning behavior of copolymers and compound systems: (**a**) P(AM-DAC-ABSM) and its supramolecular system; (**b**) P(AM-DAC-AMTU) and its supramolecular system.

The study of shear thinning behavior was via testing the viscosity of the copolymers and corresponding supramolecular systems at a shear rate of 7.34–510 s^−1^. As shown in Figure 10, the viscosity of the solution system decreased sharply with the increase of shear rate and then stabilized, which was due to the increase of shear rate entangled, curled, or even broken the polymer molecular chains, caused the significant reduced of hydrodynamic volume [48]. At a shear rate of 510 s^−1^, copolymer P(AM-DAC-ABSM) and the corresponding supramolecular system with the viscosity retention of 34.3 and 35.4 mPa·s were greater than that 26.3 and 30.2 mPa·s of P(AM-DAC-AMTU) and its supramolecular system, respectively. The appearance of this result mainly depend on the excellent shear resistance of benzene rings structure in the monomer ABSM of P(AM-DAC-ABSM), also the similar conclusions were appeared in others works [37,49]. More, the hydrogen bonding also played a significant role in the supramolecular system, showing a considerable improvement in the viscosity retention in the P(AM-DAC-AMTU) system but not obvious change in P(AM-DAC-ABSM) system. In fact, the benzene rings structure had a more remarkable contribution to shear resistance because the hydrogen bonding between molecules at high shear rate was firstly destroyed, so the shear resistance was ultimately attributed to the structure of the molecules in the system.

#### 3.3.2. Shear Recovery

**Figure 11:**
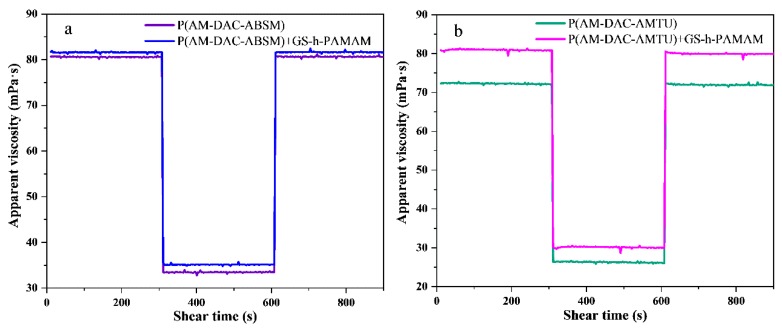
Shear recovery behavior of copolymers and compound systems: (**a**) P(AM-DAC-ABSM) and its supramolecular system; (**b**) P(AM-DAC-AMTU) and its supramolecular system.

In this section, we studied the viscosity recovery of different solution systems with a shear rate of 7.34–510 s^−1^ and then return to 7.34 s^−1^, the results were shown in Figure 11. There was a pleasant phenomenon seen where both solution systems showed great shear recovery after the change of shear rate, this result also agrees well with previous reports that the good self-healing properties of acrylamide cationic polymers [50]. Also, the viscosity retention of solution systems at high shear rate was consistent with the results in “shear thinning”. It was worth mentioning that hydrogen bonding had efficient resilience after being destroyed by strong shearing action, forming a “detachable and assemblable” supramolecular network.

#### 3.3.3. Viscoelasticity

**Figure 12:**
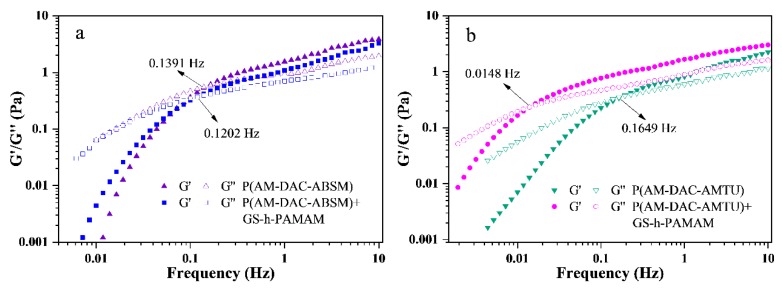
Viscoelasticity behavior of copolymers and compound systems: (**a**) P(AM-DAC-ABSM) and its supramolecular system; (**b**) P(AM-DAC-AMTU) and its supramolecular system.

To grasp the mechanical properties of copolymers and their compound systems, the viscoelastic behavior of different solution systems in the range of 10^−3^~10 Hz was determined via the oscillatory shear method (Figure 12). Both elastic modulus (G’) and viscous modulus (G”) increased gradually within the oscillation frequency range, at lower frequency, the G” of the solution systems was greater than the G’ and exhibited a viscous character, and vice versa. Universally, the reciprocal of the corresponding frequency of the curve intersection was defined as the characteristic time (1/F), the larger characteristic time, the better the viscoelasticity of the solution systems [51]. Intuitively, the addition of guanidinium salts in the copolymer P(AM-DAC-AMTU) system significantly reduced the frequency of curve intersection, meaning a great improvement in viscoelasticity. The supramolecular system was easier to form large-sized aggregates relied on hydrogen bonding and interactions between the polymer molecules, the aggregates stored high elastic modulus under stress indicating the dominance of elastic behavior [52].

## 4. Conclusions

In this work, a novel hyperbranched polyamidoamine guanidinium salts (GS-h-PAMAM) was synthesized via addition reaction of dicyandiamide and hyperbranched polyamidoamine (h-PAMAM). And two cationic acrylamide copolymers P(AM-DAC-ABSM) and P(AM-DAC-AMTU) were successfully prepared via solution radical polymerization. Then, self-assembly supramolecular systems were prepared by directly mixing GS-h-PAMAM with copolymers in aqueous solution, and the mechanism of the self-assembly process was speculated. FT-IR, NMR, and SEM were used for structural confirmation. Furthermore, there were comparative studies of the solution properties of supramolecular system and corresponding copolymer system. Excellent experimental results revealed that the supramolecular system had potential application in clay hydration inhibitors. In detail, the highest anti-swelling rate reached 96% (compound with Na^+^, K^+^, and NH_4_^+^) and 77.8% (scour five times), the highest anti-swelling retention reached 73.1% at 100°C, also with long-lasting anti-swelling performance and superior rheological properties (shear thinning, shear recovery, and viscoelasticity). More importantly, utilizing functionalized hyperbranched polyamidoamine to synthesis self-assembly supramolecular systems was an effective strategy for expanding their application fields and developing new functional materials, providing a powerful reference for the next study.

## Figures and Tables

**Table 1:** The optimum synthesis conditions for P(AM-DAC-ABSM) and P(AM-DAC-AMTU).

P(AM-DAC-ABSM)				P(AM-DAC-AMTU)			
AM:DAC	ABSM	V_50_	Tem. (°C)	AM:DAC	AMTU	V_50_	Tem. (°C)
6:4	8‰	0.3‰	65	7:3	10‰	0.3‰	55

**Table 2:** The optimal synthesis conditions for h-PAMAM and GS-h-PAMAM.

h-PAMAM			GS-h-PAMAM			
MA:DETA	Tem. (°C)	Time (h)	h-PAMAM:DCDA	pH	Tem. (°C)	Time (h)
1.2:1	140	3	1:1.5	3	80	4

## Supplementary Materials

The following are available online at https://www.mdpi.com/2073-4360/11/11/1781/s1, Figure S1: The synthetic route of ABSM; Figure S2: The (a) IR and (b) 1H NMR of ABSM; Figure S3: The synthetic route of AMTU; Figure S4: The (a) IR and (b) 1H NMR of AMTU; Figure S5: The relationship between anti-swelling rate and preparation conditions for P(AM-DAC-ABSM); Figure S6: The relationship between anti-swelling rate and preparation conditions for P(AM-DAC-AMTU); Figure S7: The FT-IR of (a) P(AM-DAC-ABSM) and (b) P(AM-DAC-AMTU); Figure S8: Relationship of ηsp/Cr (lnηr/Cr) with Cr: (a) P(AM-DAC-ABSM) and (b) P(AM-DAC-AMTU); Figure S9: The TG-DTG of (a) P(AM-DAC-ABSM) and (b) P(AM-DAC-AMTU); Table S1: The composition of copolymer P(AM-DAC-ABSM); Table S2. The composition of copolymer P(AM-DAC-AMTU); Table S3: The optimization of preparation conditions of h-PAMAM; Table S4: The optimization of preparation conditions of GS-h-PAMAM.

## References

[1] Sai W., Bin W., Junfei D. (2014). Functional Supramolecular Gels Self-Assembled by Hydrogen Bonding Among Urea-Based Gelators. Prog. Chem..

[2] Hart L.R., Nguyen N.A., Harries J.L. (2015). Perylene as an electron-rich moiety in healable, complementary π–π stacked, supramolecular polymer systems. Polymer.

[3] Schlosser F., Moos M., Lambert C. (2013). Redox-switchable Intramolecular π− π-Stacking of Perylene Bisimide Dyes in a Cyclophane. Adv. Mater..

[4] Rybtchinski B. (2013). Aqueous Supramolecular Polymers Based on Aromatic Amphiphiles: Rational Design, Complexity, and Functional Materials. Hierarchical Macromolecular Structures: 60 Years after the Staudinger Nobel Prize II.

[5] Li Y., Wang Y., Huang G. (2014). Chaotropic-anion-induced supramolecular self-assembly of ionic polymeric micelles. Angew. Chem. Int. Ed..

[6] Yuan C., Guo J., Tan M. (2014). Multistimuli responsive and electroactive supramolecular gels based on ionic liquid gemini guest. ACS Macro Lett..

[7] Evans N.H., Beer P.D. (2014). Advances in anion supramolecular chemistry: From recognition to chemical applications. Angew. Chem. Int. Ed..

[8] Miyauchi M., Kawaguchi Y., Harada A. (2004). Formation of supramolecular polymers constructed by cyclodextrins with cinnamamide. J. Incl. Phenom. Macrocycl..

[9] Castellano R.K., Clark R., Craig S.L. (2000). Emergent mechanical properties of self-assembled polymeric capsules. Proc. Natl. Acad. Sci. USA.

[10] Versteegen R.M., Van Beek D.J.M., Sijbesma R.P. (2005). Dendrimer-Based Transient Supramolecular Networks. J. Am. Chem. Soc..

[11] Liu C., Gao C., Yan D. (2007). Honeycomb-patterned photoluminescent films fabricated by self-assembly of hyperbranched polymers. Angew. Chem. Int. Ed..

[12] Zhang Y., Huang Y., Zhang J. (2015). Two unprecedented aromatic guanidines supramolecular chains self-assembled by hydrogen bonding interaction. J. Mol. Struct..

[13] Zhang Y., Huang W., Zhou Y. (2009). Complex self-assembly of hyperbranched polyamidoamine/linear polyacrylic acid in water and their functionalization. J. Phys. Chem. B.

[14] Jiang W., Zhou Y., Yan D. (2015). Hyperbranched polymer vesicles: From self-assembly, characterization, mechanisms, and properties to applications. Chem. Soc. Rev..

[15] Hu B., Pei F., Sun X. (2018). Fabrication of supramolecular hyperbranched polyamidoamine–dextran conjugates and their self-assembly in the presence of EGCG. New J. Chem..

[16] Yang W., Pan C.Y. (2009). Synthesis and fluorescent properties of biodegradable hyperbranched poly (amido amine) s. Macromol. Rapid Commun..

[17] Jiang G., Wang Y., Sun X. (2010). Facile one-pot approach for preparing fluorescent and biodegradable hyperbranched poly (amidoamine) s. Polym. Chem..

[18] -Yang W., Pan C.Y., Liu X.Q. (2011). Multiple functional hyperbranched poly (amido amine) nanoparticles: Synthesis and application in cell imaging. Biomacromolecules.

[19] Chen K.J., Wolahan S.M., Wang H. (2011). A small MRI contrast agent library of gadolinium (III)-encapsulated supramolecular nanoparticles for improved relaxivity and sensitivity. Biomaterials.

[20] Chen Y., Zhou L., Pang Y. (2011). Photoluminescent hyperbranched poly (amido amine) containing β-cyclodextrin as a nonviral gene delivery vector. Bioconjugate Chem..

[21] Pedziwiatr-Werbicka E., Milowska K., Dzmitruk V. (2019). Dendrimers and hyperbranched structures for biomedical applications. Eur. Polym. J..

[22] Mozafari M., Chauhan N.P.S. (2019). Advanced Functional Polymers for Biomedical Applications.

[23] Pérignon N., Mingotaud A.F., Marty J.D. (2004). Formation and stabilization in water of metal nanoparticles by a hyperbranched polymer chemically analogous to PAMAM dendrimers. Chem. Mater..

[24] Liu C., Gao C., Yan D. (2006). Synergistic supramolecular encapsulation of amphiphilic hyperbranched polymer to dyes. Macromolecules.

[25] Liu Z., Lin X.W., Lu R. (2019). Magnetic nanoparticles modified with hyperbranched polyamidoamine for the extraction of benzoylurea insecticides prior to their quantitation by HPLC. Microchim. Acta.

[26] Shi Y., Wang Z., Weng X. (2015). Preparation of Highly Fluorescent and pH Responsive CdTe Quantum Dots Within Dynamic Covalent Hyperbranched Polymers and Their In Vitro Application as Fluorescence Probe. Sci. Adv. Mater..

[27] Li C., Chen Y., Yang L. (2019). Tetraphenylethene decorated hyperbranched poly (amido amine)s as metal/organic-solvent-free turn-on AIE probe for specific pyrophosphate detection. Sens. Actuators B Chem..

[28] Barbey R., Perrier S. (2013). A Facile Route to Functional Hyperbranched Polymers by Combining Reversible Addition–Fragmentation Chain Transfer Polymerization, Thiol–Yne Chemistry, and Postpolymerization Modification Strategies. ACS Macro Lett..

[29] Chen Z., Zhou L., Zhang F. (2012). Multicarboxylic hyperbranched polyglycerol modified SBA-15 for the adsorption of cationic dyes and copper ions from aqueous media. Appl. Surf. Sci..

[30] Cao L. (2005). Synthesis and Functional Study of Hyperbranched Polyamidoamine Polymers. Ph.D. Thesis.

[31] Tekin N., Demirbas O., Alkan M. (2005). Adsorption of cationic polyacrylamide onto kaolinite. Microporous Mesoporous Mater..

[32] Zhang S., Qiu Z., Huang W. (2013). Characterization of a novel aluminum-based shale stabilizer. J. Pet. Sci. Eng..

[33] Hou J., Liu Y., Song G. (2016). Synthesis and Application of a New High Temperature High Performance Salt Resistant Shale Inhibitor. Drill. Fluid Complet. Fluid.

[34] Livney Y.D., Portnaya I., Faupin B. (2003). Interactions between inorganic salts and polyacrylamide in aqueous solutions and gels. J. Polym. Sci. Polym. Phys..

[35] Balaban R.D.C., Vidal E.L.F., Borges M.R. (2015). Design of experiments to evaluate clay swelling inhibition by different combinations of organic compounds and inorganic salts for application in water base drilling fluids. Appl. Clay Sci..

[36] Sarsenbekuly B., Kang W., Fan H. (2017). Study of salt tolerance and temperature resistance of a hydrophobically modified polyacrylamide based novel functional polymer for EOR. Colloids Surf. A.

[37] Jia H., Chen H. (2018). Using DSC technique to investigate the non-isothermal gelation kinetics of the multi-crosslinked Chromium acetate (Cr_3_^+^)-Polyethyleneimine (PEI)-Polymer gel sealant. J. Pet. Sci. Eng..

[38] Xiao X., Kong D., Qiu X. (2015). Shape-memory polymers with adjustable high glass transition temperatures. Macromolecules.

[39] Tan B., Thomas N.L. (2016). A review of the water barrier properties of polymer/clay and polymer/graphene nanocomposites. J. Membr. Sci..

[40] Ayirala S., Yousef A. (2015). A state-of-the-art review to develop injection-water-chemistry requirement guidelines for IOR/EOR projects. SPE Prod. Oper..

[41] Boek E.S., Coveney P.V., Skipper N.T. (1995). Monte Carlo molecular modeling studies of hydrated Li-, Na-, and K-smectites: Understanding the role of potassium as a clay swelling inhibitor. J. Am. Chem. Soc..

[42] Lin L., Luo Y., Li X. (2019). Synthesis of Diblock Polyampholyte PAMPS-b-PMAPTAC and Its Adsorption on Bentonite. Polymers.

[43] Ma J., Cui P., Zhao L. (2002). Synthesis and solution behavior of hydrophobic association water-soluble polymers containing arylalkyl group. Eur. Polym. J..

[44] Zhong H., Qiu Z., Zhang D. (2016). Inhibiting shale hydration and dispersion with amine-terminated polyamidoamine dendrimers. J. Nat. Gas Sci. Eng..

[45] Khodja M., Canselier J.P., Bergaya F. (2010). Shale problems and water-based drilling fluid optimisation in the Hassi Messaoud Algerian oil field. Appl. Clay Sci..

[46] Nojiri S., Yamada H., Kimata S. (2016). Supramolecular polypropylene with self-complementary hydrogen bonding system. Polymer.

[47] Stahl G.A., Schulz D.N. (2012). Water-Soluble Polymers for Petroleum Recovery.

[48] Miao L., Guo H., Zuckermann M.J. (1996). Conformation of polymer brushes under shear: Chain tilting and stretching. Macromolecules.

[49] Chen W., Chen J., Liu L. (2013). Effects of chain stiffness on conformational and dynamical properties of individual ring polymers in shear flow. Macromolecules.

[50] Sato K., Nakajima T., Hisamatsu T. (2015). Phase-separation-induced anomalous stiffening, toughening, and self-healing of polyacrylamide gels. Adv. Mater..

[51] Ferry J.D. (1980). Viscoelastic Properties of Polymers.

[52] Farajzadeh R., Andrianov A., Zitha P.L.J. (2009). Investigation of immiscible and miscible foam for enhancing oil recovery. Ind. Eng. Chem. Res..

