# Supplementary material for: Self-Assembly Supramolecular Systems Based on Guanidinium Salts Modified Hyperbranched Polyamidoamine and Cationic Acrylamide Copolymers
Source: Polymers (Basel). 2019 Oct 31;11(11):1781. doi: 10.3390/polym11111781 (PMC6918400; doi:10.3390/polym11111781)
Supplement: Supplementary file 1 [file polymers-11-01781-s001.pdf]

Supplementary material

Self-assembly Supramolecular Systems Based on Guanidinium Salts Modified Hyperbranched Polyamidoamine and Cationic Acrylamide Copolymers

Qi Wu [1,3], Shaohua Gou [1,2,*], Yumei Fei [1], Xiaoyan Yang [1], Mengyu Liu [1] and Jinglun Huang [3]

[1] College of Chemistry and Chemical Engineering, Southwest Petroleum University, Chengdu 610500, China; 201722000166@swpu.stu.edu.cn (Q.W.); 201621000271@swpu.stu.edu.cn (Y.F.); 201721000259@swpu.stu.edu.cn (X.Y.); 201722000183@swpu.stu.edu.cn (M.L.)

[2] State Key Laboratory of Oil and Gas Reservoir Geology and Exploitation, Southwest Petroleum University, Chengdu 610500, China

[3] Institute of Chemical Materials, China Academy of Engineering Physics, Mianyang 621900, China; jinglunhuang@163.com (J.H.)

* Correspondence: shaohuagou@swpu.edu.cn (S.G.)

1. Preparation and characterization of monomer ABSM

Preparation of monomer ABSM. Into a 250 mL single-necked round bottom flask with a magnetic stir bar, triethylamine (20 mL) was added into p-toluenesulfonyl chloride (12.6 g, 0.066 mol) with 50 mL CH_2Cl_2. Then allylamine (4.14 g, 0.073 mol) dissolved with 20 mL CH_2Cl_2 was slowly dropped into the flask, the entire process controlled system temperature at 0-5°C, completed, the reaction was warmed to 25°C and maintained for 6 h. After the reaction, the mixture was washed with 1% hydrochloric acid solution, 1% sodium hydroxide solution and saturated sodium chloride solution, respectively, then dried over anhydrous sodium sulfate, filtered and evaporated, a white solid powder allylbenzenesulfonamide ABSM was obtained in 92.3% yield.

^{1}H NMR (400 MHz, $CDCl_3$) δ (ppm): 7.73 (d, 2H), 7.29 (d, 2H), 5.79–5.62 (m, 1H), 5.10 (dd, 2H), 4.65 (s, 1H), 3.55 (s, 2H), 2.40 (s, 3H).

FT-IR (cm^{-1}): 3250(vs), 2940(w), 2850(m), 1600(vw), 1430(s), 1330(vs), 1160(vs),780(s).

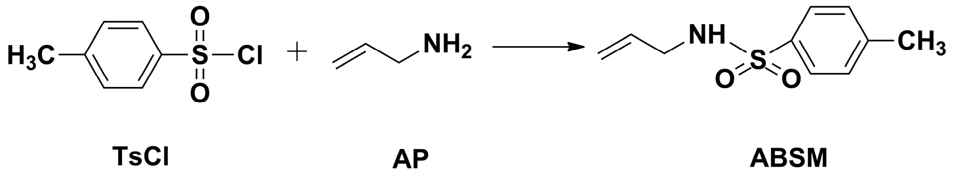

Figure S1. The synthetic route of ABSM.

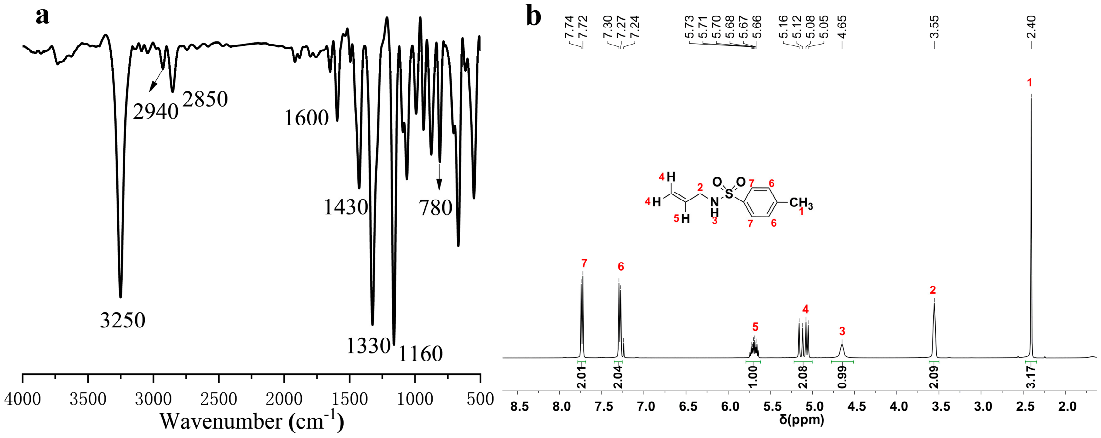

Figure S2. The (a) IR and (b) ^{1}H NMR of ABSM.

2. Preparation and characterization of monomer AMTU

Preparation of monomer AMTU. Methyl isothiocyanate (7.21 g, 0.10 mol), triethylamine (30 mL) and 30 mL acetonitrile were added into a 250 mL three-necked round bottom flask with a magnetic stir bar, condenser and thermometer. After completely dissolved, allylamine (6.24 g, 0.11 mol) was slowly dropped into the mixture, then raised the temperature to 60°C. After refluxed for 7 h, the crude product was washed repeatedly with diethyl ether, filtered, evaporated, a white powder product 1-allyl-3-methylthiourea AMTU was obtained in 78.1% yield.

^{1}H NMR (400 MHz, D_2O) δ (ppm): 7.72 (s, 1H), 7.30-7.32 (m, 1H), 5.57-5.59 (m, 1H), 5.12-5.16 (m, 4H), 2.43 (s, 3H).

FT-IR (cm^{-1}): 3350(s), 3080(m), 2980(w), 2850(m), 1330(vs), 1195(vs).

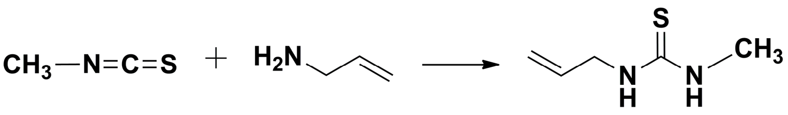

MTC AP AMTU

Figure S3. The synthetic route of AMTU.

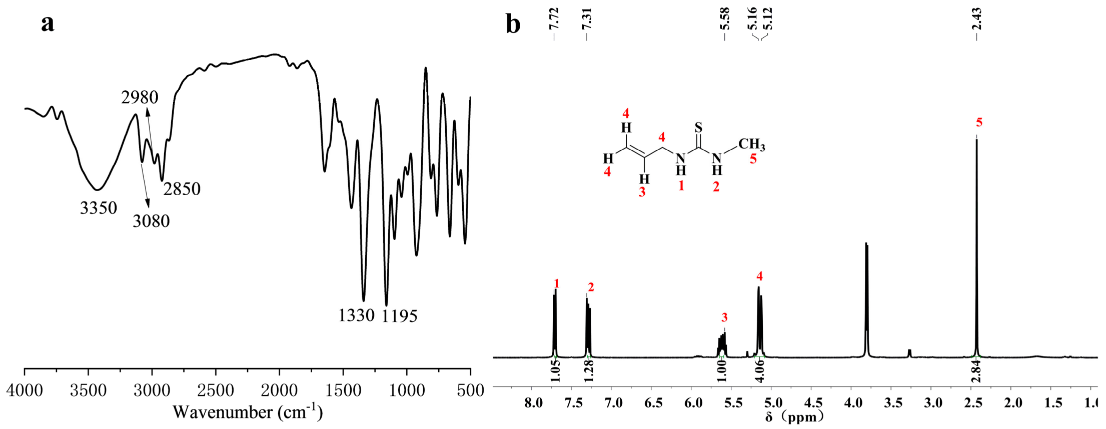

Figure S4. The (a) IR and (b) ^{1}H NMR of AMTU.

3. Rate of anti-swelling determination method

The rate of anti-swelling of polymer solution to bentonite was determined by centrifugation method, which according to Chinese oil and gas industry standard SY/T 5971-1994 "Performance evaluation method of clay stabilizer for water injection", the details was as follows: a graduated 10.0 mL centrifuge tube with 0.50 g dried bentonite sample, 10.0 mL polymer solution was added and stirred evenly, let it stand at a constant temperature for 2 h. Then the sample was centrifuged at 1500 r/min for 15 min, and the swelling volume of bentonite was recorded as V_1. The swelling volumes of bentonite in pure water V_2 and in kerosene V_0 were measured via the same method, the rate of anti-swelling of the polymer solution was calculated by the [Eq. (S1)].

$$B = (V_2 - V_1) / (V_2 - V_0) \times 100\% \qquad \text{(S1)}$$

wherein B was the rate of anti-swelling of the polymer solution; V_0 was the swelling volume of bentonite in kerosene, mL; V_1 was the swelling volume of bentonite in pure water, mL; and V_2 was the swelling volume of bentonite in the polymer solution, mL. According to the results of previous experiments, The V_1 and V_0 value of the bentonite used in this paper (montmorillonite) were 3.2 and 0.6, respectively.

4. Optimization, characterization and performance of copolymers P(AM-DAC-ABSM) and P(AM-DAC-AMTU)

4.1 Optimization process of preparation conditions

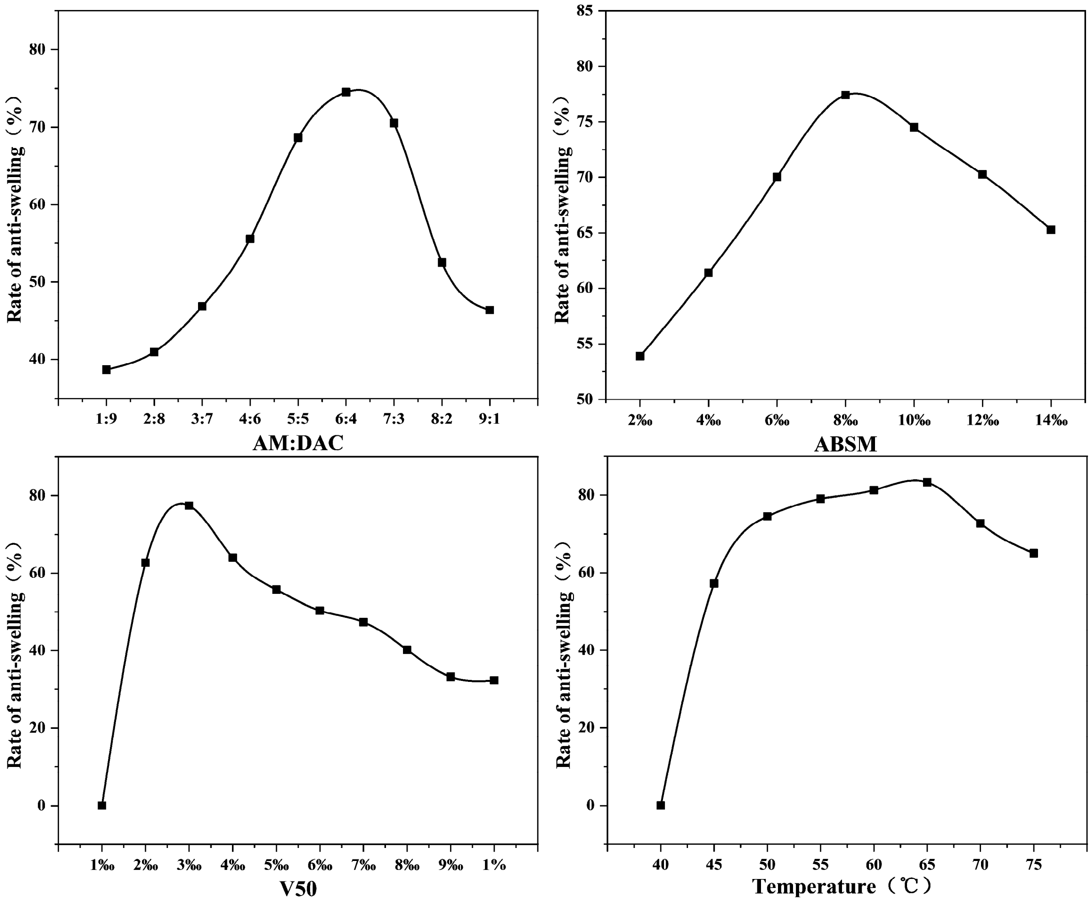

Figure S5. The relationship between anti-swelling rate and preparation conditions for P(AM-DAC-ABSM).

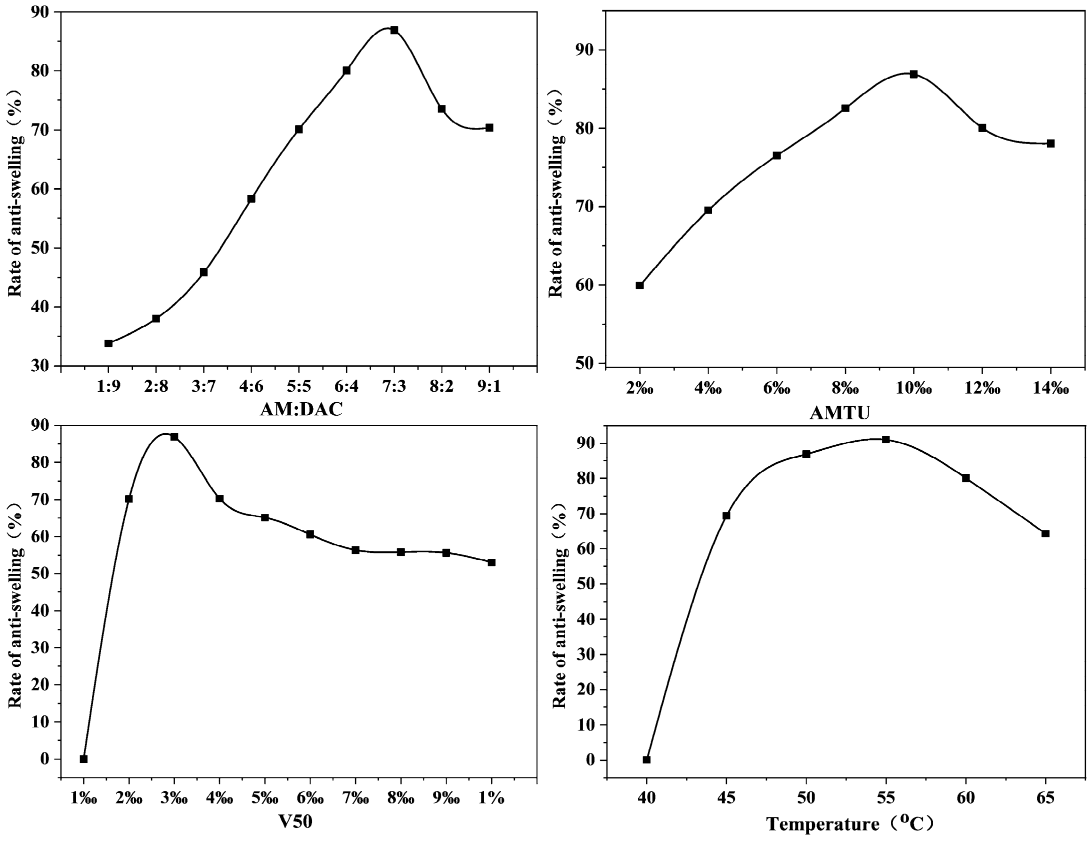

Figure S6. The relationship between anti-swelling rate and preparation conditions for P(AM-DAC-AMTU).

4.2 Characterization

4.2.1 FT-IR

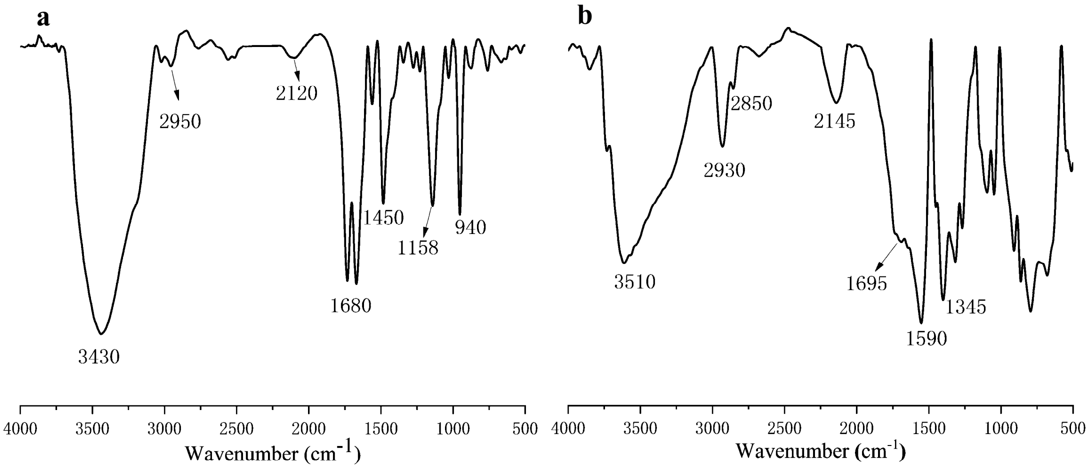

Figure S7. The FT-IR of (a) P(AM-DAC-ABSM) and (b) P(AM-DAC-AMTU).

4.2.2 Composition determination

Due to the long molecular chain length and complex structure of the copolymers, conventional characterization methods were difficult to determine the structure. In

order to determine whether the functional monomer successfully entered the copolymer backbone and determined the composition of the copolymer, the composition of the copolymers were determined by high performance liquid chromatography. The AM, DAC, and ethanol used to purify the polymer were measured, respectively, and the test conditions were AM: ODS column, V (methanol): V (water) = 9:1, 210 nm, 40 °C, 1.0 mL/min; DAC: ODS column, V (acetonitrile): V (water) = 9:1, 210 nm, 40 °C, 1.0 mL/min. The standard curve for each monomer was determined as: $A_{AM} = 4.4655 \times 10^7C + 517452.3$; $A_{DAC} = 4102.98C + 1138.1$. Further, the conversion ratio of each component and its proportion in the copolymer were calculated from the [Eq. (S2)], and the results were shown in Table S1 and S2.

$$\alpha = \frac{W - V\frac{AC_0}{A_0}}{W} \times 100\% \tag{S2}$$

wherein α was the monomer conversion rate (%); W was the monomer raw charge quality (g); A was the monomer peak area in the chromatogram of the ethanol used for purifying the polymer; C_0/A_0 was the reciprocal of the slope of the monomer standard curve; V was the total volume (L) of ethanol used to purify the polymer.

Table S1. The composition of copolymer P(AM-DAC-ABSM)[a]

Entry	Initial feed ratio (wt%)			Final composition (wt%)		
	AM	DAC	ABSM	AM	DAC	ABSM
P(AM/DAC/ABSM)	60.0	39.0	1.0	63.2	36.0	0.8

[a]All data were the average of three measurements with an error of ±0.1.

Table S2. The composition of copolymer P(AM-DAC-AMTU)[a]

Entry	Initial feed ratio (wt%)			Final composition (wt%)		
	AM	DAC	AMTU	AM	DAC	AMTU
P(AM/DAC/AMTU)	69.0	30.0	1.0	71.8	27.3	0.9

[a]All data were the average of three measurements with an error of ±0.1.

4.2.3 Intrinsic Viscosity Measurement

Intrinsic viscosity [η] was decided with a NCY automatic Ubbelohde capillary viscometer (0.55 mm) (Shanghai Sikeda scientific instruments Inc., Shanghai, China)

at 30 ± 0.1°C. The copolymers were dissolved and diluted to five different concentrations (C=1.0, 0.67, 0.50, 0.33, 0.25 mg/L) with 1 mol/L NaCl solution. The flux times of the copolymer solutions had to be accurate to ± 0.2 s. The specific viscosity and intrinsic viscosity were calculated via the following [Eq. (S3 and S4)].

$$\eta_{sp} = \frac{t - t_0}{t_0} \quad \text{(S3)}$$

$$[\eta] = \lim_{c \to 0} \frac{\eta_{sp}}{c} \quad \text{(S4)}$$

wherein η_{sp} was the specific viscosity of polymer; c was pure polymer solution concentration, mg/L; t_0 was flux time of 1 mol/L NaCl brine, s; and t was flux time of polymer brine solution, s.

The specific calculations of the measurement of intrinsic viscosity were according to the [Eq. (S3 and S4)]. From Figure S8, the intrinsic viscosity values of copolymers P(AM-DAC-ABSM) and P(AM-DAC-AMTU) were 706 mL/g and 734 mL/g, respectively.

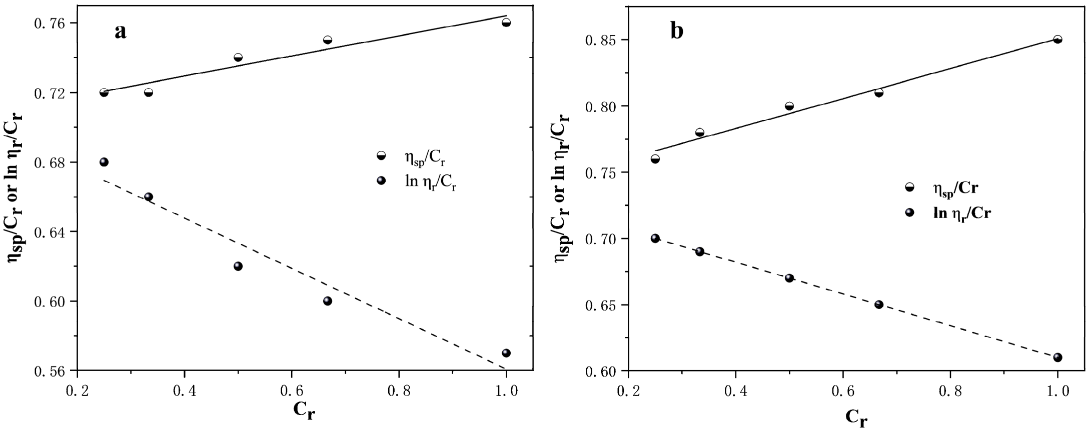

Figure S8. Relationship of ηsp/Cr (lnηr/Cr) with Cr: (a) P(AM-DAC-ABSM) and (b) P(AM-DAC-AMTU).

4.3 Performance (TG-DTG)

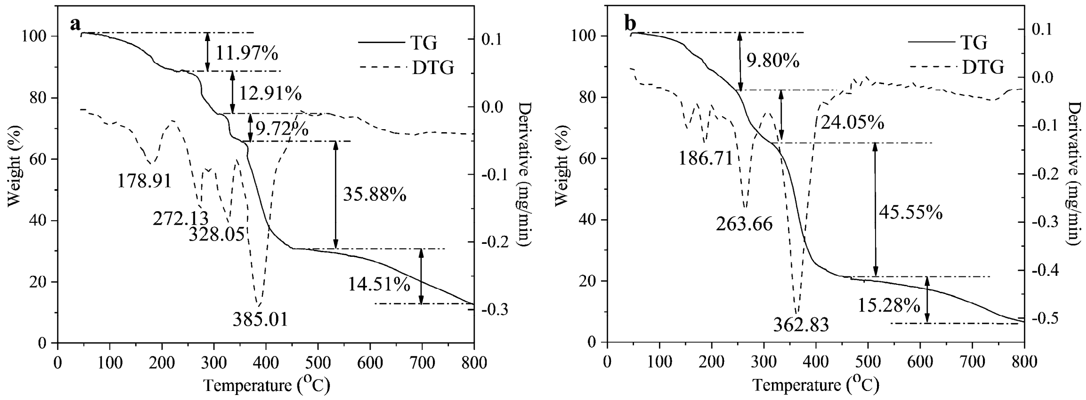

Figure S9. The TG-DTG of (a) P(AM-DAC-ABSM) and (b) P(AM-DAC-AMTU).

5. The optimization of preparation conditions of h-PAMAM and GS-h-PAMAM

Table S3. The optimization of preparation conditions of h-PAMAM[a]

Entry	MA:DETA	Temperature (°C)	Time (h)	η[b] (mPa·s)	Yield (%)
1	1:1	150	3	125.8	92.5
2	1.2:1	150	3	156.9	94.2
3	1.5:1	150	3	Insoluble	/
4	1.75:1	150	3	Insoluble	/
5	2:1	150	3	Insoluble	/
6	2.5:1	150	3	100.4	91.1
7	1.2:1	60	3	64.8	75.2
8	1.2:1	80	3	87.1	81.6
9	1.2:1	100	3	122.8	87.5
10	1.2:1	120	3	144.7	91.3
11	1.2:1	140	3	157.3	94.7
12	1.2:1	150	1	95.5	71.9
13	1.2:1	150	2	128.3	84.6
14	1.2:1	150	4	157.5	94.8
15	1.2:1	150	5	157.2	94.5

[a]All data were the average of three measurements with an error of ± 0.1; [b]Apparent viscosity of h-PAMAM.

Table S4. The optimization of preparation conditions of GS-h-PAMAM[a]

Entry	h-PAMAM:DCDA	Temperature (°C)	pH	Time (h)	Yield (%)
1	1:1	90	3	4	45.1
2	1:1.2	90	3	4	64.2
3	1:1.5	90	3	4	87.1
4	1:1.8	90	3	4	85.8
5	1:2	90	3	4	85.3
6	1:2.5	90	3	4	81.1
7	1:1.5	60	3	4	55.2
8	1:1.5	70	3	4	82.8
9	1:1.5	80	3	4	91.5

10	1:1.5	100	3	4	90.8
11	1:1.5	110	3	4	91.1
12	1:1.5	80	1	4	58.9
13	1:1.5	80	2	4	79.7
14	1:1.5	80	4	4	No product
15	1:1.5	80	5	4	No product
16	1:1.5	80	2	2	86.8
17	1:1.5	80	2	3	89.2
18	1:1.5	80	2	5	91.0
19	1:1.5	80	2	6	91.7

[a]All data were the average of three measurements with an error of ±0.1.

6. Test methods on rate of anti-swelling after different scouring times

The basic method for testing the rate of anti-swelling was according to the steps in "3. Rate of anti-swelling determination method". After centrifugation, the supernatant was poured out, 10.0 mL of deionized water was added, stirred evenly, and allowed to stand for 2 h. The rate of anti-swelling was tested via the same method, repeated the above steps 4 times and recorded the results for each time.

7. Test methods on cuttings recovery rate

The cuttings recovery rate of different solutions system was determined according to the Chinese oil and gas industry standard SY/T 5613-2000 "Physical and Chemical Properties Test Method for Mud Shale", the details was as follows: grinded the core, through a 40 mesh sieve, dried at 105°C for 8 h, then cooled to room temperature. Weighed 50.0 g (accurate to 0.1 g) of dried cuttings and filled it in a high temperature tank with 350.0 mL different solutions, rolling in a roller furnace at 80°C for 16 h. After the hot rolling was completed, took out the high temperature tank and cooled to room temperature, poured the sample into a 40 mesh sieve and wetted it with tap water for 1 min. The remaining cuttings samples were placed at 105°C oven for 6 h, cooled and weighed, and recorded as M_1, the first cuttings recovery rate R_1 was calculated via [Eq. (S5)]. The cuttings were again subjected to the same method as in the high temperature tank containing 350.0 mL of deionized water, and M_2 was measured according to the above procedure, and the second cuttings recovery rate R_2 was calculated via the [Eq. (S6)].

$$R_1 = M_1 / M_0 \times 100\% \quad \text{(S5)}$$

$$R_2 = M_2 / M_0 \times 100\% \quad \text{(S6)}$$

wherein R_1 was the first cuttings recovery rate, %; M_1 was the residual mass after the first hot rolling of the cuttings, g; R_2 was the second cuttings recovery rate, %; M_2 was the residual mass after the second hot rolling of the cuttings, g; M_0 was the initial mass of cuttings, 50.0 g.

8. Test methods on rheological behavior of solution system

Polymer (0.2 wt%) of rheological behavior was conducted by a HAAKE MARS III rheometer (HAAKE, Germany) at an appropriate temperature.

8.1 Shear thinning and shear recovery test

Shear thinning of 2000 mg/L polymer solution was measured via a rotation rheometer (Haake RheoStress 6000, Germany) with a shear rate range of 7.34-510 s^{-1} at 30 ± 0.1°C, the shear recovery was performed with stepped shear rate 7.34-510-7.34 s^{-1} under the same conditions as others.

8.2 Viscoelasticity

Viscoelasticity of polymer solution (2000 mg/L) was obtained via measuring the elastic modulus (G') and viscous modulus (G") with particular stress (1 Pa) at 30°C in the frequency scanning range: 0.01-10 Hz. The behavior of the viscoelasticity was described via [Eq. (S7 and S8)]).

$$G'(\omega) = G\frac{\omega^2\lambda^2}{1+\omega^2\lambda^2} \quad \text{(S7)}$$

$$G''(\omega) = G\frac{\omega\lambda}{1+\omega\lambda} \quad \text{(S8)}$$

wherein G was stress relaxation modulus, λ was relaxation time, and ω was angular frequency.
